# Domain-Specific vs. General-Purpose Large Language Models in Orthodontics: A Blinded Comparison of AlimGPT, GPT-4o, Gemini, and Llama

**DOI:** 10.3390/dj14040219

**Published:** 2026-04-08

**Authors:** Sertaç Aksakallı, Bilgin Giray, Çağrı Temel

**Affiliations:** 1 Department of Orthodontics, Istanbul Gelisim University, Istanbul 34310, Turkey; 2 Department of Orthodontics, Cyprus Health and Social Sciences University, Guzelyurt 99750, Cyprus; giray.bilgin@gmail.com; 3 Hezarfen LLC, Mountain View, CA 34990, USA; cagritemel34@gmail.com

**Keywords:** artificial intelligence, language model, reliability, orthodontic planning

## Abstract

**Objective**: The application of artificial intelligence (AI) in orthodontics has evolved rapidly in recent years, encompassing areas such as diagnosis, treatment planning, and patient management, and AlimGPT is an AI-based tool that provides treatment options based on data and algorithms. **Methods**: Fourteen different orthodontic questions were asked to each model, and answers were analyzed. This study aimed to compare AlimGPT with GPT-4o, Gemini, and Llama using standardized tests to evaluate the quality of information provided, including the Likert scale, modified DISCERN (mDISCERN), and modified Global Quality Score (mGQS). **Results**: Significant differences were detected for reliability (χ^2^ = 15.267, *p* = 0.0016) and usefulness (χ^2^ = 20.557, *p* = 0.0001). Post hoc tests showed AlimGPT > Gemini and Llama for reliability and AlimGPT > GPT-4o, Gemini, and Llama for usefulness. mDISCERN was significant overall (χ^2^ = 11.047, *p* = 0.0115), but no pairwise contrast met adjusted significance; mGQS showed no significant differences (χ^2^ = 7.071, *p* = 0.0697). Inter-rater agreement was moderate-to-good for reliability (ICC = 0.710, 95% CI 0.60–0.80) and usefulness (ICC = 0.729, 95% CI 0.63–0.82), moderate for mGQS (ICC = 0.596, 95% CI 0.47–0.71), and poor-to-moderate for mDISCERN (ICC = 0.435, 95% CI 0.30–0.58). **Conclusions**: In this blinded, within-subjects experiment, the domain-specific model (AlimGPT) received higher clinician ratings for usefulness and, for reliability, exceeded two general baselines. Differences in mGQS were not detected. Expanding the number of raters, increasing item diversity or integrating updated baselines would be beneficial.

## 1. Introduction

Artificial intelligence (AI) is transforming orthodontics, assisting orthodontists with diagnosis, developing treatment plans, and tracking patients, and it is allowing for more accurate treatments and better-quality results for patients. With the progress of artificial intelligence, it will be more integral to orthodontic treatment in the future, which will provide more effective, efficient, and convenient treatments to all parties; thus, it will significantly influence orthodontics [1].

Applications within the realm of LLMs are very broad, including healthcare, education, and customer service, and these LLMs can exhibit human-like writing and intelligent conversation. Thus, there is very high demand to apply these models in a large number of verticals, such as orthodontics, because a number of orthodontic applications based on large language models have been designed to assist clinicians with orthodontic diagnosis, treatment planning, and prediction [2,3,4]. One example of such an application is AlimGPT, an orthodontic language model that provides orthodontic knowledge and offers orthodontic prediction in the form of a conversational agent, so with this model, orthodontists can communicate with patients and ask questions about their orthodontic conditions. The model can then respond with predicted outcomes for various treatment options; however, the author, who holds recognized certifications—academic training in artificial intelligence—explicitly warns against the use of such models because large language models are not doctors [5,6]. They are designed to assist; therefore, the impact of these tools on physicians’ decisions, treatment processes, and patient outcomes must be understood to ensure their appropriate application in restorative dentistry.

We evaluated LLMs answering orthodontic questions and case scenarios, evaluating accuracy and relevance, and our goal was to determine whether LLMs understand orthodontic principles and can provide actionable clinical advice. There are no prior studies comparing orthodontically trained models to general-purpose global LLMs; therefore, this study aims to fill the gap in existing research.

AI is of critical significance in orthodontics because of the following: the application of AI in orthodontics is revolutionizing the practice by increasing diagnostic accuracy and treatment planning, and integration is leading to better patient outcomes as well as more efficient treatment procedures [7,8,9]. Despite increased deployment of AI within orthodontic care, currently, most prevalent AI models—even the most robust large language models—are still not specifically tailored in orthodontics and will thus demonstrate limited capacity for complex decision-making at deeper levels. Current general large language models (LLMs) still represent an enormous shortfall in capability related to responses over very intricate scenarios within orthodontics, like proper treatment plans, biomechanics, options of appliances used, and indeed, tailored personal patient care [10]. Therefore, orthodontic practice requires skilled, domain-specialist AI systems individually created to address the complex nuances of orthodontic procedure and thus accelerate diagnostic accuracy, clinical efficiency, and patient benefit. The intention behind this paper is to respond to this crucial gap in requirement by comparing a specialized orthodontic large language model, AlimGPT, against current state-of-the-art general-purpose LLMs and to provide evidence towards the superior decision support and clinical usefulness of specializing AI systems in orthodontics. It is important to note that this is a preliminary study, and while the findings are promising, further extensive research with larger datasets and diverse methodologies is warranted to comprehensively validate AlimGPT’s or similar orthodontic LLMs’ capabilities.

**Figure 1:**
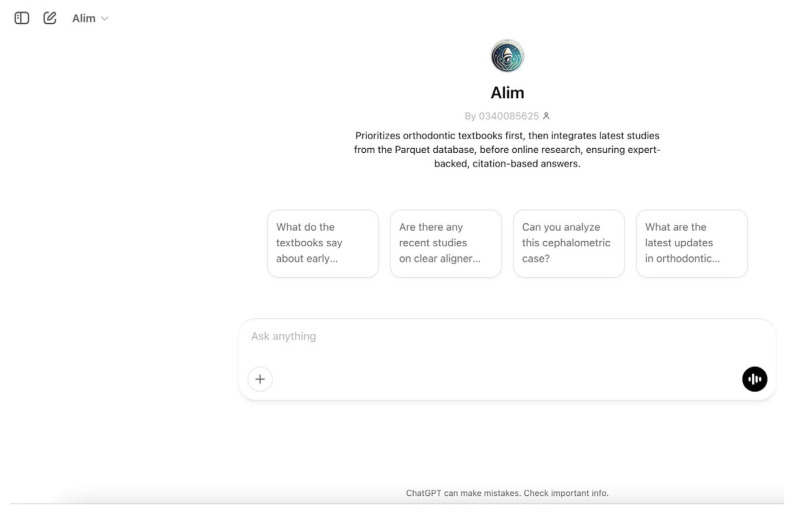
The figure shows the user interface of AlimGPT, the large language model examined in this study.

The development process of AlimGPT incorporated crucial human-in-the-loop feedback and clinical validation from orthodontic experts, facilitating reinforcement learning that aligns precisely with evidence-based protocols. This targeted language modeling approach underpins AlimGPT’s capability to support precision diagnostics, advanced treatment planning, and continuous education within the field of orthodontics (Figure 1).

Our primary hypothesis was that the domain-specific model (AlimGPT) would achieve higher scores in reliability and usefulness compared to general-purpose models. We tested these using a blinded, within-subjects design with orthodontist raters and a content-validated 14-item prompt set.

The purpose of this study was to determine whether AlimGPT provides higher-quality and more clinically reliable orthodontic information compared with general-purpose large language models (GPT-4o, Gemini, and Llama) using standardized evaluation tools such as Likert scales, the modified Global Quality Score (mGQS), and the modified DISCERN instrument (mDISCERN).

## 2. Materials and Methods

In accordance with the PICOS framework, the population comprised four orthodontists with ≥4 years of clinical experience who rated answers to 14 orthodontic prompts. The intervention (Index test) was the domain-specific large language model AlimGPT, and the comparators were three general-purpose models: GPT-4o, Gemini, and Llama. The outcomes were clinician-rated reliability and usefulness (anchored Likert scales, with usefulness additionally assessed by the modified Global Quality Score, mGQS) and trustworthiness (assessed by the modified DISCERN, mDISCERN). The study design was a rater-blinded, within-subjects, repeated-measures experiment in which each rater evaluated responses from all four models to every prompt, enabling direct model comparisons while controlling for rater and item effects.

The study was conducted in accordance with the Declaration of Helsinki. Since it did not involve human or animal data, ethics committee approval was not required. This study assessed the performance of AI models and did not involve any clinical trials on human patients or use any private patient data. The ‘human participants’ were expert raters performing professional evaluation tasks, not subjects of experimentation. This study did not involve patients or patient data. All evaluations were performed by licensed orthodontists assessing anonymized AI-generated text. According to the institutional and national guidelines, ethics committee approval was not required for this type of study. Thus, IRB approval is exempted by the institutional review board in accordance with local regulations for non-clinical expert evaluations.

The 14 evaluation prompts were developed by the authors, all trained orthodontists, to reflect clinically and academically relevant questions commonly encountered in orthodontic practice. Prompt content was informed by core orthodontic textbooks, established clinical knowledge, and the prior literature evaluating large language models in dentistry. The structure and content of these prompts were inspired from previous studies evaluating large language models in dentistry [11,12], as well as core orthodontic textbooks and established clinical guidelines. This adaptation ensured that the prompts covered a balanced spectrum of topics, including clear aligner therapy, biomechanics, and temporary anchorage devices (TADs), consistent with validated testing protocols in the literature. Two experienced orthodontists reviewed the prompt list in order to confirm that it was clear, logical, and provided equal coverage across both the academic and clinical domains. All prompts were finalized before model querying and were applied identically to all evaluated models, with no adaptive modification or performance-based refinement during the study. This approach was intended to support standardized comparisons and reduce potential selection bias. To minimize the risk of data leakage or model-specific overfitting, prompt selection and wording were finalized prior to any model querying and were not adapted based on model performance. All evaluated models were queried using identical, fixed prompts under the same conditions. There was no specific retrieval, tuning, or repetitive refinement for the prompts during the evaluation phase.

**Statistical Analysis:** All statistical analyses were performed in Python v3.10 using the PyCharm environment (version 2023.2.2), scipy, statsmodels, pandas and MedCalc^®^ v 19.7 Statistical Software version 19.7.2 (MedCalc Software Ltd., Ostend, Belgium; https://www.medcalc.org, accessed on 7 November 2025). Prior to data collection, the plan was to use an a-priori, simulation-based power analysis tailored to the planned mixed-effects model (Outcome~Model + (1|Rater) + (1|Question)). Assuming a moderate standardized fixed effect of model (≈0.30–0.40 SD), random-intercept SDs for rater, and question of 0.30–0.50, k = 4 models, 14 items, and 4 raters with complete repeated measures; Monte-Carlo simulations (α = 0.05, two-sided) indicated approximately 0.80 power to detect an omnibus model effect using a Type-III F-test with small-sample df correction.

To minimize order and expectancy effects, randomization for the presentation of model outputs at the rater × prompt level using a computer-generated sequence; within each prompt, all four models appeared once per rater in a counterbalanced order. To preserve blinding, responses were exported into a uniform template that removed all metadata (e.g., model names, logos, links) and standardized typography; models were presented as Model A–D, and the allocation key was withheld from investigators and raters until analyses were complete. Before data collection, the exclusion rules were specified: (i) non-answers (empty output, API error/refusal, or content not addressing the prompt), (ii) duplicated outputs after >2 technical retries, (iii) responses in a language other than English or containing disallowed content, and (iv) incomplete rater forms.

We asked all models 14 questions (Table 1, including 2 sections with different types of questions: clinical and academic, case management, treatment plan, latest studies, biomechanics and materials) and then we compared their answers to AlimGPT v1.0. This helped us see how good each model was because we picked questions that covered many parts of orthodontics. This included treatment plans, patient care, and new orthodontic tech, and 4 experienced orthodontists (with minimum 4 years of experience) red the answers and scored them using a scale. No responses were excluded. In cases of equivalent ratings, tied scores were retained without adjustment. All model outputs were assessed based on criteria that were determined in advance. Responses were rated with three validated scoring tools based on the prior literature: the Likert scale for clinical relevance, the modified DISCERN tool for reliability, and the modified Global Quality Score (mGQS) for general quality. In the current study, responses were rated with three different scales:

- Likert Scale—A 7-point scale that allows evaluators to rate their perceptions and satisfaction regarding the answers. The Likert scale was used in our study in order to evaluate the overall quality and clinical adequacy of the responses (Score 1: critical failure, Score 7: flawless accuracy).
- mDISCERN (Modified DISCERN) Scale—A modified version of the DISCERN instrument, which was originally created to assess the quality of information available on the internet about medicine. In this study, the mDISCERN scale was applied to assess the reliability of the LLM-generated responses and their use of credible sources. (Score 1: No source, fake or hallucinated, Score 5: High quality).
- mGQS (Modified Global Quality Score)—A scoring system designed to evaluate the overall quality and presentation integrity of health-related online information. In this study, the mGQS scale was used to evaluate the usefulness and presentation quality of the responses. (Score 1: Poor, Score 5: Excellent).

This study was conducted using the Likert scale for reliability and use, the mDISCERN scale, and the modified Global Quality Score, and these tests were used to assess the quality of medical information. The use of these tests helped to improve and optimize the current research; thus, they helped to compare between different AI systems like AlimGPT. This is very important because it makes this study more effective and standardized. Addressing this need, AlimGPT, a novel domain-specific AI assistant meticulously engineered for orthodontic applications, was developed and tested. AlimGPT was developed using the OpenAI GPT-4 Turbo API. To reduce hallucinations and guarantee domain specificity, a Retrieval-Augmented Generation (RAG) model was utilized. The knowledge base comprised twenty-five orthodontic textbooks and a database of peer-reviewed articles published over the last 5 years. Upon reception of a prompt, the system retrieved the most relevant chunks from the vector database. Built upon OpenAI’s GPT-4 Turbo architecture, AlimGPT’s training involved an extensive dataset exceeding 300 million tokens, meticulously curated from core orthodontic textbooks and comprehensive clinical reference materials. In order to keep its relevance and maintain rigor, the model regularly incorporates insights from a dedicated repository of recent peer-reviewed research, specifically in the areas of biomechanics, aligner therapy, and AI-assisted treatment planning.

## 3. Results

Table 2 summarizes the mean (±standard deviation) and median (interquartile range) values for reliability, usefulness, mDISCERN, and mGQS parameters across the four evaluated LLM models (AlimGPT, GPT-4o, Gemini 2.0, and Llama 3).

Inter-rater reliability among orthodontists was assessed using the intraclass correlation coefficient (ICC), calculated as a two-way random-effects model with absolute agreement (Table 3). Orthodontists demonstrated moderate-to-good agreement for Reliability (ICC = 0.710, 95% CI: 0.60–0.80) and Usefulness (ICC = 0.729, 95% CI: 0.63–0.82), moderate agreement for mGQS (ICC = 0.596, 95% CI: 0.47–0.71), and poor-to-moderate agreement for mDISCERN (ICC = 0.435, 95% CI: 0.30–0.58).

Differences among the four evaluated LLM models (AlimGPT, GPT-4o, Gemini 2.0, and Llama) were analyzed using Friedman’s tests, with pairwise post hoc Wilcoxon’s signed-rank tests employing Bonferroni’s corrections (Table 4). Significant differences were detected in reliability (χ^2^ = 15.267, *p* = 0.0016) and usefulness (χ^2^ = 20.557, *p* = 0.0001). Post hoc analysis showed that AlimGPT received higher reliability scores than Gemini and Llama and higher usefulness scores than GPT-4o, Gemini, and Llama. Though mDISCERN showed a significant overall difference (χ^2^ = 11.047, *p* = 0.0115), the following pairwise comparisons did not achieve statistical significance. No significant differences among the LLMs were identified for mGQS (χ^2^ = 7.071, *p* = 0.0697).

**Figure 2:**
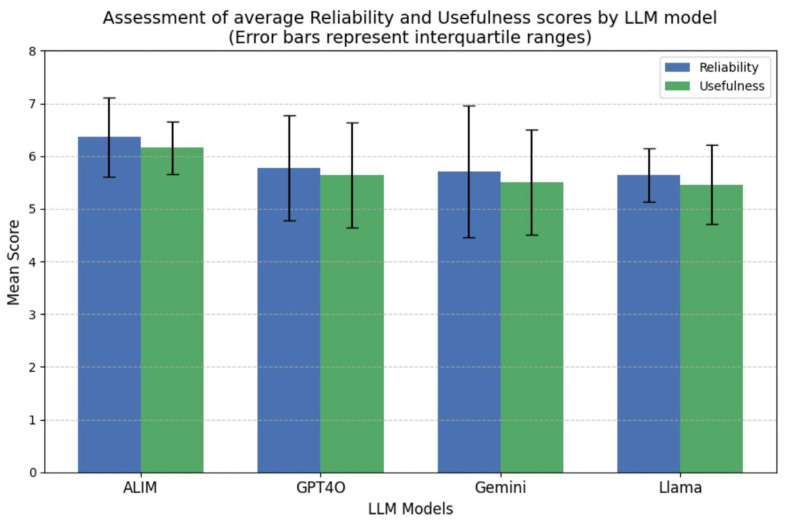
Assessment of mean reliability and usefulness scores across the four evaluated large language models (AlimGPT, GPT-4o, Gemini, and Llama).

In Figure 2, bars indicate mean scores, and error bars represent interquartile ranges (IQRs), illustrating variability among the questions evaluated by the orthodontists. AlimGPT demonstrated the highest mean scores in both reliability (6.36 ± 0.41) and usefulness (6.16 ± 0.34), significantly outperforming Gemini and Llama in reliability and GPT-4o, Gemini, and Llama in usefulness (*p* < 0.05, Friedman’s test with Bonferroni-corrected Wilcoxon’s post hoc comparisons).

## 4. Discussion

The use of AI has changed the world of orthodontics, and AI offers better precision in diagnosis and treatment planning. It is more efficient than old methods because AI is now used in more diagnostic imaging and predictive analytics, and this allows orthodontists to use personalized treatment planning. With the introduction of novel AI tools such as AlimGPT, the efficacy of these tools needs to be evaluated, and their integration into current workflows also needs to be considered [5,6]. Clinically, domain-specific LLMs may serve as adjunctive decision-support tools for orthodontists by assisting with preliminary information synthesis, patient education, and academic training while not replacing clinician judgment.

There are many diverse usages of AI in dentistry today, including tasks such as symptom assessment and personalized treatment suggestions, efficient appointment scheduling, comprehensive planning and evaluation of various dental treatments, as well as detailed x-ray analysis to assist dentists in making informed decisions [7,8,9].

Large language models may improve patient care in dentistry, and this includes diagnosis of disease and treatment plans [10]. In the current study, the inter-rater agreement was moderate-to-good for reliability (ICC: 0.710), usefulness (ICC: 0.729) and mGQS (ICC: 0.596) but poor for mDISCERN (ICC: 0.435), which is in line with Tanaka et al. [11]. They reported poor inter-rater reliability (Fleiss’ kappa statistic of 0.004) for ChatGPT-4o responses assessing clear aligners and orthodontic digital imaging, and hence, our study shows better inter-rater reliability than Tanaka et al. [11]. This may indicate that the agreement is improved among raters with orthodontic experience because our results suggest that the level of expertise of the raters can affect the inter-rater agreement; thus, this is an important consideration for future studies.

Although blinded ratings were employed, explicit controls on prompt finalization and non-adaptive querying were implemented to further mitigate potential concerns related to prompt-induced bias, data leakage, or overfitting. Orthodontists reveal that while our trained model, AlimGPT, achieved the highest scores overall, it is important to note that all the language models performed satisfactorily when a user asked about some definitions or explanations. To enhance reproducibility, representative anonymized outputs from each model for selected prompts are provided. While general orthodontic questions involving definitions or basic explanations were satisfactorily answered by most evaluated LLMs, AlimGPT demonstrated a distinct advantage in detailed clinical decision-making contexts such as “How is posterior open bite closure managed in aligner therapy?”, “Which wire and bracket combination is recommended for torque control?”, “ The adult patient has Class II division 2 malocclusion. The molars are in half unit Class II. She has lower anterior facial height and decreased FMA angle. What is your orthodontic treatment plan?”. Recent studies indicate that the use of a specific orthodontic LLM may enhance clinical practice and treatment planning. Specifically, AlimGPT provided comprehensive, step-by-step recommendations in orthodontic treatment planning, including aligners—such as specifying precise arch-wire sequences required at each stage or deeper cephalometric analysis–explanations—thus showcasing its superior integration of up-to-date, evidence-based orthodontic research and clinical guidelines. This specific ability may possibly lessen doubts about diagnoses and make the clinic run more smoothly, leading to better results for patients and more informed decisions in the clinic. They showcased a thorough grasp of the subject, indicating that each LLM is competent and capable. Also, the orthodontists reported that the ability of these models to adjust to different situations is very useful in real-life clinical situations. When looking at the quality and trustworthiness of what was made, our study showed that the answers from AlimGPT had a quality between moderate and high. This finding aligns with the work of Balel [13], who identified moderate quality in outputs produced by ChatGPT in the context of maxillofacial surgery using the GQS evaluation framework. Additionally, the research conducted by Dursun and Bilici Geçer [14] corroborated these results, as they discerned that ChatGPT-4 produced responses characterized by moderate reliability and commendable quality, reflected in a mean GQS score of 3.8 ± 0.62. This evidence is in close agreement with our own mGQS findings, which reported a mean score of 4.09 ± 0.19 for GPT-4o.

Our mDISCERN mean scores (4.04–4.39) were notably higher than those reported by Kılınç and Mansız [12] (DISCERN mean: 2.38 ± 0.27–3.04 ± 0.06) in their evaluation of ChatGPT-4 orthodontic content. This disparity can likely be attributed to differences in evaluator specialties or professional knowledge, suggesting that the evaluators’ backgrounds significantly influence subjective scoring. While Kılınç and Mansız [12] reported high mDISCERN reliability (mean ± SD: 4.1 ± 0.7 and 3.8 ± 0.6), our study observed lower inter-rater reliability, which implies that mDISCERN can be subjective and may possess inherent ambiguity. As a result, the evaluator’s knowledge and the personal nature of the judgment are very important factors [15,16].

Overall, we found that commonly used big LLMs were able to generate useful responses to both clinical and academic queries in orthodontics, and we acknowledge that with the rapid advancement of natural language processing and machine learning, future iterations of AI systems will likely produce even more precise and relevant responses [17,18,19]. Ethically, users should be aware that the use of AI in orthodontics must comply with strict rules to protect patient privacy and data security because AI-generated responses can offer valuable insights, but they should never be relied upon to replace the clinical judgement of an orthodontist. Clinical decisions about patient management should always be made by trained professionals, with AI serving as a supplementary tool rather than a definitive source; thus, the phrase “ChatGPT-4o can make mistakes. Check important info” should be a reminder to people that they need to verify information generated by AI, especially in fields like orthodontics, where precision and accuracy are very important.

One of the biggest concerns in the digital age is the risk of misinformation or misinterpretation of data, and this underscores the importance of rigorous validation processes and close collaborations between AI developers and orthodontic practitioners; therefore, such collaborations are crucial to ensure that AI algorithms are not only accurate but also aligned with current orthodontic practices and guidelines [20,21].

Large language models sometimes give wrong answers, even if they are trained on a lot of data. Reasons for errors can include the formulation of the question and the response, because question formulation can lead to a different response. Hallucination, or making up results, is a particularly problematic issue when the responses are expected to be accurate, such as in medical applications, it can therefore produce significant problems. LLMs can produce plausible responses but are also prone to producing both fabricated content and outdated responses; thus, a review of ChatGPT-related studies reported that the majority of studies expressed concerns regarding the risk of responses being incorrect [22]. In our study, reliability (mDISCERN) and usefulness (mGQS and Likert scale) were used as practical indicators of these risks. Models that scored lower in reliability often displayed tendencies consistent with hallucination or factual inaccuracy, such as providing references that did not match the content or offering outdated clinical recommendations. Conversely, AlimGPT—a domain-specific model trained with curated orthodontic sources—achieved the highest reliability and usefulness scores. This suggests that domain-specific training and constrained knowledge boundaries can reduce the likelihood of hallucination by narrowing the model’s output space to vetted, specialty-relevant information. In real-world applications, this focused method not only reduces the chances of creating incorrect material but also improves how clinically relevant the suggestions made by AI are. The discrepancy between the significant superiority of AlimGPT in ‘usefulness’ and the lack of significant difference in ‘mGQS’ highlights a critical distinction. While general-purpose LLMs (GPT-4o, Gemini) are highly proficient in generating grammatically coherent and well-structured text (high mGQS), they may lack the specific clinical nuance required for actionable treatment planning. AlimGPT’s higher ‘usefulness’ scores suggest that while the form of the answer was similar across models, the clinical content provided by the domain-specific model was deemed more applicable by the orthodontists.

They also expressed concerns that it might provide inaccurate information, lack originality and fabricate responses, and its responses are limited by the data it was trained on. It does not work well with rare or new medical problems, but it can be applied to help with some medical tasks like report writing or saving time and effort. Several publications demonstrated that it performed well on medical examinations, so this suggests that it can be used in educational and clinical decision-making assistance [23]. However, the examination also revealed that the LLM occasionally provided brief or incorrect responses because it either provided insufficient information or omitted relevant information when asked about rare medical conditions, such as temporomandibular joint inflammation [24].

Artificial intelligence has permeated various domains of dentistry, ranging from diagnosis to treatment planning and patient management. In orthodontics, it holds the promise of revolutionizing our therapeutic strategies. It can lead to more personalized and efficient treatment options; thus, it can greatly improve the field of orthodontics, and in our recent study, we demonstrated that AlimGPT could achieve promising results on orthodontic data; therefore, this technology could be highly beneficial in the field of orthodontics. The integration of similar orthodontic LLMs will help to streamline the diagnostic processes and enhance treatment planning. But one limitation for the current study should be underscored; the low inter-rater agreement for mDISCERN suggests that evaluating ‘trustworthiness’ is inherently subjective. Consequently, any inferences drawn about this metric must be interpreted with caution. Utilizing sophisticated algorithms, such models have the potential to enhance the analysis of patient data, thus facilitating better clinical results and personalized treatment. In the near future, we believe that these kinds of LLMs will continue to improve (Alim will continue as well) as new techniques in machine learning are developed. For tasks similar to those assessed in this research, it is possible that a domain-specific system offers responses that clinicians perceive as more usable and reliable; however, such deployment should be cautious and under clinician oversight, in line with current best practices. Integrating specific LLMs like AlimGPT with existing orthodontic practices could streamline workflows and enhance decision-making processes, thus ensuring that practitioners can deliver the highest standard of care [10,24].

This study shows that even though general-purpose AI models are available, they might not have the accuracy needed for complex orthodontic decision-making. The clinical significance of these findings lies in suggesting that domain-specific fine-tuning, as implemented in AlimGPT, may reduce the likelihood of clinically irrelevant or unsupported content in treatment planning tasks, particularly in areas such as biomechanics and appliance selection, as reflected by higher clinician-rated usefulness and reliability scores. **Limitations of the Study:** There are some limitations to be considered while interpreting the results of this study. First, although the within-subjects design allowed for direct comparisons, the sample size of four orthodontist evaluators is relatively small. While a power analysis indicated sufficient power for the primary outcomes, a larger and more diverse panel of raters from different institutions would enhance the generalizability of the results and reduce potential subjective bias. Second, the evaluation was limited to 14 specific orthodontic prompts. These questions dealt with many clinical and academic topics but may not reflect the full complexity of everyday orthodontic practice. Consequently, the performance of the models on untested, highly niche, or extremely complex scenarios remains unknown. Third, the inter-rater reliability for the mDISCERN scale was found to be poor-to-moderate (ICC = 0.435), suggesting that evaluating the “trustworthiness” of AI outputs remains inherently subjective and difficult to standardize among human raters, and given the poor-to-moderate ICC for mDISCERN, results related to trustworthiness should be interpreted cautiously and were not used to support the primary conclusions. Finally, the study was conducted at a specific point in time using specific versions of the models (e.g., GPT-4o, Gemini, Llama). Given the rapid iteration cycle of large language models, the relative performance rankings observed in this study may shift as newer versions are released. A further important constraint is the difference in architecture between the experimental and control groups. The domain-specific model (AlimGPT) utilized a Retrieval-Augmented Generation (RAG) framework, granting it access to a curated external knowledge base of textbooks and peer-reviewed articles. In contrast, the general-purpose models (GPT-4o, Gemini, and Llama) were evaluated in their standard ‘out-of-the-box’ states, relying solely on their pre-trained parametric memory. Consequently, it is difficult to discern whether AlimGPT’s superior performance stems from the domain-specific fine-tuning or simply the access to external reference materials. To ensure a more rigorous evaluation of domain specificity, future research should implement a control group where general-purpose models are also equipped with the same external knowledge base (e.g., GPT-4o + RAG) to isolate the impact of the model architecture versus the retrieval mechanism.

**Recommendations for Future Research:** Based on the results and limitations of this research, several research suggestions for future studies are proposed. Future studies should seek to include larger panels of evaluators across multiple centers to increase statistical robustness and eliminate individual rater bias. It is also recommended to significantly expand the prompt library to include hundreds of diverse scenarios, potentially drawing from real-world, anonymized patient cases rather than standardized textbook questions. Additionally, since orthodontics is a heavily visual discipline relying on radiographs and photographs, future studies should evaluate the “multi-modal” capabilities of these models—specifically their ability to analyze cephalometric images or intraoral scans alongside text. Furthermore, research into the “explainability” of these domain-specific models is warranted; clinicians need to understand not just what the treatment plan is but why the AI recommends it, citing specific evidence-based guidelines. Finally, prospective clinical trials assessing the actual impact of AI assistance on chair-side efficiency and treatment outcome quality would provide the most definitive evidence of clinical utility.

## 5. Conclusions

Among the evaluated large language models, the domain-specific model AlimGPT received higher clinician-rated usefulness scores and, for reliability, outperformed two general-purpose models. While overall differences in trustworthiness (mDISCERN) were observed, pairwise comparisons did not reach statistical significance, and inter-rater agreement for this metric was modest, indicating that conclusions related to trustworthiness should be interpreted cautiously. Within the limitations of the evaluated metrics, prompt set, and rater sample, these findings suggest that domain-specific training may enhance the clinical applicability of AI-generated orthodontic information, while continued clinician oversight remains essential.

## Figures and Tables

**Table 1:** List of prompts used in the study.

Category	Prompt ID	Standardized Prompt Content
Academic/Biomechanics	Q1	Compare orthognathic surgery outcomes and management between clear aligners and traditional fixed appliances.
	Q3	Define the physiological limits of orthodontic forces and their biological implications.
	Q5	Which arch-wire-and-bracket combinations are recommended for effective torque control?
	Q7	Describe the biomechanics and design of segmental arches using mini-screws (TADs).
	Q9	Describe the biomechanics and methods for lower anterior intrusion using fixed appliances.
	Q10	What are the optimal force magnitude and protocols for face mask therapy in maxillary protraction?
	Q11	Describe the clinical protocol and anatomical considerations for the placement of Infrazygomatic Crest (IZC) screws.
	Q12	Define “Class IV malocclusion” in the context of orthodontic classifications.
Clinical Management	Q2	What are the treatment strategies for managing unilateral Class II malocclusion using fixed appliances?
	Q4	How is posterior open bite managed during clear aligner therapy?
	Q6	What are the specific periodontal risks associated with orthodontic treatment in a 35-year-old adult patient?
Case Scenarios	Q8	Case 1: A 12-year-old male presents with anterior open bite, maxillary constriction, and Class II molar/canine relationship. Outline a comprehensive treatment plan.
	Q13	Case 2: An adult female patient presents with Class II Division 2 malocclusion, a half-unit Class II molar relationship, and a decreased FMA angle. Outline the orthodontic treatment plan.
	Q14	Case 3: A 10-year-old female patient presents with a persistent tongue thrust habit. What are the recommended therapeutic interventions?

**Table 2:** Mean (±SD) and median (IQR) scores per parameter by LLM (descriptive statistics).

Parameter	LLM	Mean ± SD	Median (IQR)
Reliability (1–7)	AlimGPT	6.36 ± 0.41	6.38 (6.00–6.75)
	GPT-4o	5.77 ± 0.73	6.00 (5.00–6.00)
	Gemini	5.70 ± 0.75	5.88 (5.00–6.25)
	Llama	5.64 ± 0.50	5.75 (5.50–6.00)
Usefulness (1–7)	AlimGPT	6.16 ± 0.34	6.13 (6.00–6.50)
	GPT-4o	5.64 ± 0.69	5.75 (5.00–6.00)
	Gemini	5.50 ± 0.81	5.50 (5.00–6.00)
	Llama	5.46 ± 0.65	5.50 (5.00–5.75)
mDISCERN (1–5)	AlimGPT	4.39 ± 0.23	4.38 (4.25–4.50)
	GPT-4o	4.04 ± 0.27	4.00 (4.00–4.25)
	Gemini	4.27 ± 0.22	4.25 (4.00–4.50)
	Llama	4.04 ± 0.24	4.00 (4.00–4.25)
mGQS (1–5)	AlimGPT	4.27 ± 0.11	4.25 (4.25–4.31)
	GPT-4o	4.09 ± 0.19	4.13 (4.00–4.25)
	Gemini	4.20 ± 0.18	4.25 (4.00–4.25)
	Llama	4.15 ± 0.23	4.25 (4.00–4.25)

**Table 3:** Inter-rater reliability results (intraclass correlation coefficient—two-way random-effects model).

Parameter	ICC (95% CI)	Interpretation
Reliability	0.710 (0.60–0.80)	Moderate-to-Good
Usefulness	0.729 (0.63–0.82)	Moderate-to-Good
mDISCERN	0.435 (0.30–0.58)	Poor-to-Moderate
mGQS	0.596 (0.47–0.71)	Moderate

**Table 4:** Statistical comparisons of LLMs (analyzed using Friedman’s test with Bonferroni-corrected Wilcoxon’s post hoc tests).

Parameter	Friedman Test	Post Hoc Significant Differences (Wilcoxon, Bonferroni-Corrected)
Reliability	χ^2^ = 15.267; *p* = 0.0016	AlimGPT > Gemini (*p* = 0.0295), AlimGPT > Llama (*p* = 0.0475)
Usefulness	χ^2^ = 20.557; *p* = 0.0001	AlimGPT > GPT-4o (*p* = 0.0425), AlimGPT > Gemini (*p* = 0.0294), ALIM > Llama (*p* = 0.0103)
mDISCERN	χ^2^ = 11.047; *p* = 0.0115	No significant pairwise differences after correction
mGQS	χ^2^ = 7.071; *p* = 0.0697	No significant differences

## Data Availability

The data presented in this study are available on request from the corresponding author.
